# The effects of inositol supplementation on lipid profiles among patients with metabolic diseases: a systematic review and meta-analysis of randomized controlled trials

**DOI:** 10.1186/s12944-018-0779-4

**Published:** 2018-05-24

**Authors:** Reza Tabrizi, Vahidreza Ostadmohammadi, Kamran B. Lankarani, Payam Peymani, Maryam Akbari, Fariba Kolahdooz, Zatollah Asemi

**Affiliations:** 1 Health Policy Research Center, Institute of Health, Student Research Committee, Shiraz University of Medical Sciences, Shiraz, Iran; 2 Research Center for Biochemistry and Nutrition in Metabolic Diseases, Kashan University of Medical Sciences, Kashan, Islamic Republic of Iran; 3 Health Policy Research Center, Shiraz University of Medical Sciences, Shiraz, Iran; 4 Indigenous and Global Health Research, Department of Medicine, University of Alberta, Edmonton, Canada

**Keywords:** Inositol, Lipid profiles, Metabolic diseases, Meta-analysis

## Abstract

**Background:**

Several studies have evaluated the effect of inositol supplementation on lipid profiles among population with metabolic diseases; however, the findings are controversial. This review of randomized controlled trials (RCTs) was performed to summarize the evidence of the effects of inositol supplementation on lipid profiles among population with metabolic diseases.

**Methods:**

Relevant RCTs studies were searched in Cochrane Library, EMBASE, MEDLINE, and Web of Science until October 2017. Two researchers assessed study eligibility, extracted data, and evaluated risk of bias of included primary studies, independently. To check for the heterogeneity among included studies Q-test and I_2_ statistics were used. Data were pooled by using the random-effect model and standardized mean difference (SMD) was considered as summary of the effect size.

**Results:**

Overall, 14 RCTs were included into meta-analysis. Pooled results showed that inositol supplementation among patients with metabolic diseases significantly decreased triglycerides (SMD − 1.24; 95% CI, − 1.84, − 0.64; *P* < 0.001), total- (SMD − 1.09; 95% CI, − 1.83, − 0.55; *P* < 0.001), and LDL-cholesterol levels (SMD − 1.31; 95% CI, − 2.23, − 0.39; *P* = 0.005). There was no effect of inositol supplementation on HDL-cholesterol levels (SMD 0.20; 95% CI, − 0.27, 0.67; *P* = 0.40).

**Conclusions:**

Inositol supplementation may result in reduction in triglycerides, total- and LDL-cholesterol levels, but did not affect HDL-cholesterol levels among patients with metabolic diseases. Additional prospective studies regarding the effect of inositol supplementation on lipid profiles in patients with metabolic diseases are necessary.

## Background

There are numerous primary and secondary causes of hypertriglyceridemia and *hypercholesterolemia*, such as genetics, lifestyle and diet, especially obesity and physical inactivity, diseases, including metabolic syndrome (MetS), hyperinsuliemia, diabetes mellitus, and renal disease [1, 2]. The incidence of elevated triglycerides levels varies by age, and is consistently higher in men than women [3]. Previous studies have demonstrated that hypertriglyceridemia is often correlated with insulin resistance, and chronic disease, including type 2 diabetes mellitus (T2DM) and chronic kidney disease [3, 4]. In addition, increased levels of total- and LDL-cholesterol are associated with an increased risk of atherosclerotic cardiovascular diseases (CVD) [5].

Various approaches are suggested to control blood glucose and lipid profiles, including lifestyle modification and pharmacological therapy [6, 7]. One such emerging potential intervention is inositols supplementation (e.g. myo-inositol (MI) and di-chiro inositol (DCI)) which showed insulin-mimetic properties efficient in lowering post-prandial blood glucose [8]. *MI* is the primary biologically active form of inositol which has a six-carbon sugar alcohol and is one of nine biologically significant isomers of hexahydroxycyclohexane [9]. DCI is also active and recognized as an important messenger in insulin signal transduction [9]. Inositols are present in many foods, especially fresh fruits and vegetables, beans, grains, and, nuts. Inositols have been mainly used in treating several pathologies including polycystic ovary syndrome (PCOS) [10], MetS [11], and gestational diabetes (GDM) [12]. The favorable effects of a dietary supplement of inositols have been studied in the last years [13, 14]. In a meta-analysis study conducted by Pundir et al. [15], inositols supplementation could improve menstrual cycles, ovulation and metabolic changes in patients with PCOS. Moreover, Giordano et al. [11] demonstrated that MI supplementation significantly improved systolic and diastolic blood pressure, insulin resistance, cholesterol, and triglycerides levels in postmenopausal women with MetS. In lean subjects with PCOS, DCI supplementation decreased circulating levels of insulin, serum androgens, and improved some of the metabolic abnormalities such as increased blood pressure and hypertriglyceridemia [16].

Many randomized controlled trials (RCTs) have been conducted to determine whether inositol supplementation has a causal effect on lipid profiles among population with metabolic diseases. We aimed to systematically review the present evidence on the effect of inositol supplementation on lipid profiles in RCTs and to summarize the available findings in a meta-analysis, if possible.

## Methods

### Search strategy

Relevant studies were systematically searched from online databases PubMed, EMBASE, Web of Science, and Cochrane Library databases, until October 2017 by using the following MeSH and text words keywords: patients [“metabolic disease” OR “Mets” OR “diabetes” OR “T2DM” OR “GDM” OR “PCOS”], intervention (“MI” OR “inositol” OR “DCI” OR “DCI” AND “supplementation” OR “administration” OR “taking” OR “intake”), and outcomes [“triglycerides (TG)” OR “total-cholesterol” OR “low-density lipoprotein cholesterol (LDL-cholesterol)” OR “high-density lipoprotein cholesterol (HDL-cholesterol)”]. International Standard Randomized Controlled Trial Number Register and Meta-register for RCTs were searched for finding ongoing and archived RCTs. The search study was conducted by two independent researchers. References cited in the selected studies were manually searched for additional relevant articles. Our search was restricted to studies published in English.

### Selection criteria

The eligibility criteria were: human RCTs, patients with metabolic diseases, the administration of inositol supplements, studies that were compared the inositol group with control or placebo group, and studies that have reported mean changes of lipid profiles including triglycerides, total-, LDL-, and HDL-cholesterol along with standard deviation for the intervention and control groups. Studies that did not report mean changes of lipid profiles, along with standard deviation (SD) for the intervention and control groups, the abstracts of seminars without full text, case reports, and studies that did not obtain the minimum required score of quality assessment process were excluded.

### Quality assessment

Two authors have performed data extraction (VO and MA) and quality assessment (RT and MA), independently when there was disagreement between them were resolved by a third author (ZA). We used the Cochrane Collaboration risk of bias tool to assess the quality of the included RCTs based on information on the following domains: randomization generation, allocation concealment, blinding, analyses with intention to treat, withdrawals and drop-outs data, selective reporting, other sources of bias.

### Statistical methods

RevMan software (Cochrane Review Manager, version 5.2) and STATA version 12.0 (Stata Corp., College Station, TX) were used for data analyses. Heterogeneity was evaluated through the Cochran (Q) and I-squared tests (I_2_). Given the existing heterogeneity between studies, when I_2_ exceeds 50% or *P* < 0.05, the random-effect model was used; otherwise, the fixed-effect model was applied. Inverse variance method and Cohen statistics were used for estimation of standardized mean difference (SMD) and 95% CI for verifying the outcomes behavior of each study group (intervention/control). Sensitivity analyses also undertook in the trials one by one to evaluate the reliability of the pooled mean difference. In addition, the Cochrane Collaboration Risk of Bias tool was used to assess the methodological quality of the RCTs. Potential publication bias was assessed through visual inspection of funnel plots and quantitatively assessed using Egger’s tests.

## Results

### Characteristics of included studies

**Fig. 1:**
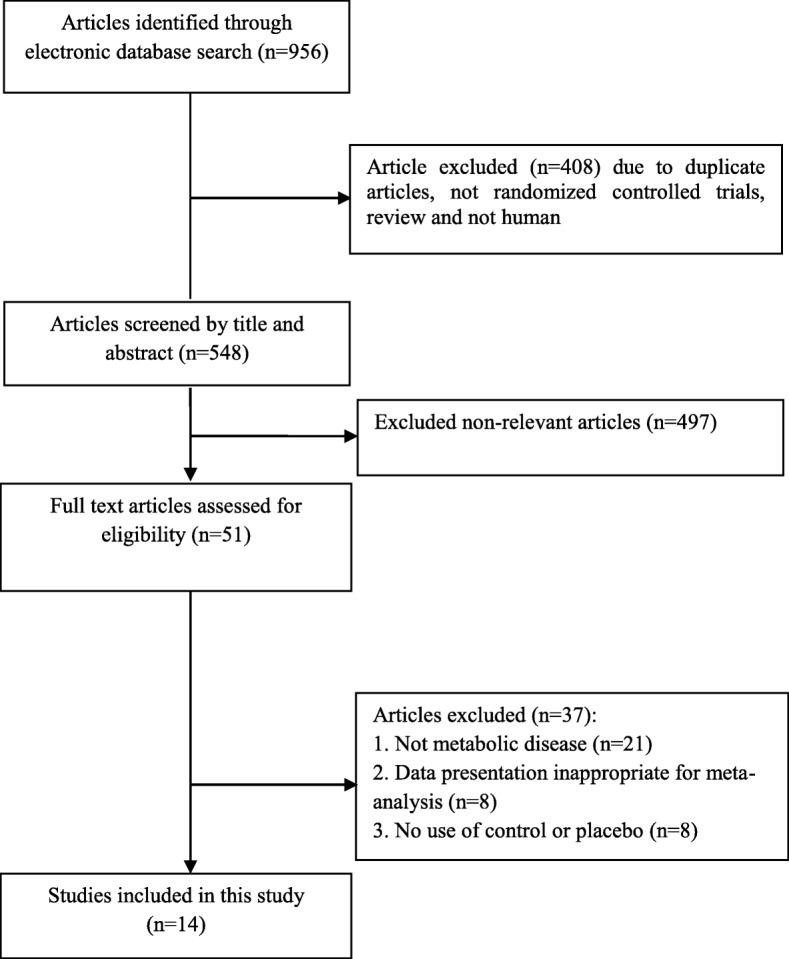
Literature search and review flowchart for selection of studies

From 956 potential citations, 14 articles were included in our meta-analysis. The flow chart of step by step details for study identification and selection is illustrated in Fig. 1. Eight studies were used double-blind design [16–23], four were randomized placebo-controlled trial design [11, 24–26], and two were randomized controlled trial design [27, 28]. Seven trials evaluated the effects of inositol on lipid profiles among patients with PCOS [16, 19, 20, 22, 23, 27, 28] and others were evaluated the effects of inositol on other metabolic diseases [11, 17, 18, 21, 24–26]. Twelve studies have reported the effects of inositol on triglycerides, 11 on total cholesterol, five on LDL-cholesterol, and ten on HDL-cholesterol levels. These articles have been published from 1999 to 2017. Eleven studies were conducted in Italy [11, 17–20, 22, 24–28], two in Venezuela [16, 23], and one in Republic of Korea [21]. The mean age of included participants was ranged between 22.79 ± 4.13 to 61.7 ± 7.74 yeras. The duration of intervention ranged between 6 weeks to 12 months. The dosage of inositol (including its derivatives such as MI and/or DCI) varied from 688 mg to 4000 mg/day. Table 1 shows characteristics of the included trials.

**Table 1:** Characteristics of included studies

Authors (Ref)	Publication year	Sample size (control/intervention)	Country/population	Intervention (name and daily dose)	Duration	Presented data	Age (y) (control, intervention)	Results
Malvasi et al. [17]	2017	35/34	Italy/overweight pregnant	138 mg MI + 550 mg DCI	60 days	TG, TC, LDL-C, HDL-C	32.31 ± 5.99, 32.35 ± 4.62	Decreased TC, LDL-C, HDL-C, TG
Cianci et al. [27]	2015	20/26	Italy/PCOS	1000 mg DCI + 600 mg a-lipoic acid	180 days	TG, TC, HDL-C	23.8 ± 2.5	Increased HDL-C
Malvasi et al. [18]	2014	24/24	Italy/overweight pregnant	2000 mg MI + 400 mg DCI + 400 μg FA + 10 mg manganese	60 days	TG, TC, LDL-C, HDL-C	31.58 ± 5.66, 32.2 ± 5.46	Decreased TC, LDL-C, HDL-C and TG
D’Anna et al. [24]	2014	24/26	Italy/postmenopausal women with MetS	2000 mg MI + 30 mg cocoa polyphenols + 80 mg soy isoflavones	6 months	TG, HDL-C	55.5 ± 4.8, 56.3 ± 3.8	Decreased TG
Capasso et al. [25]	2013	78/77	Italy/postmenopausal women with MetS	4000 mg Inositol + a-lipoic acid	6 months	TG, TC, HDL-C	58.2 ± 5.6, 57.71 ± 7.9	Decreased TG, increased HDL-C
Santamaria et al. [26]	2012	40/40	Italy/postmenopausal women with MetS	4000 mg MI (2 g b.i.d)	12 months	TG, TC, HDL-C	55 ± 3.2, 55.6 ± 3.2	Decreased TG, TC, increased HDL-C
Giordano et al. [11]	2011	40/40	Italy/postmenopausal women with MetS	4000 mg MI (2 g b.i.d)	6 months	TG, TC, HDL-C	55 ± 3.2, 55.6 ± 3.2	Decreased TG, TC, increased HDL-C
Minozzi et al. [28]	2011	75/80	Italy/PCOS	4000 mg MI + 400 μg FA + combined OCP	12 months	TG, TC, LDL-C, HDL-C	29.4 ± 4.1, 28.8 ± 3.8	Decreased LDL-C, increased HDL-C
Costantino et al. [19]	2009	19/23	Italy/PCOS	4000 mg MI + 400 μg FA	12–16 weeks	TG, TC	27.1 ± 1.4, 28.8 ± 1.5	Decreased TG, TC
Gerli et al. [20]	2007	47/45	Italy/PCOS	4000 mg MI + 400 μg FA	14 weeks	TG, TC, LDL-C, HDL-C	29.7 CI [28.5–30.9], 29.0 CI [27.1–30.9]	Increased HDL-C
Kim et al. [21]	2005	15/15	Republic of Korea/T2DM	1200 mg (600 mg twice) pinitol (D-3-O-methyl-Chiro-Inositol)	13 weeks	TG, TC, LDL-C, HDL-C	61.7 ± 7.74, 59.9 ± 12	Decreased TC, LDL-C, Increased HDL-C
Gerli et al. [22]	2003	39/26	Italy/PCOS	200 mg (100 mg twice) inositol	14 weeks	TG, TC, LDL-C, HDL-C	29.2 CI [27.5–30.7], 28.6 CI [26.9–30.3]	Increased HDL-C
Iuorno et al. [16]	2002	10/10	Venezuela/PCOS	600 mg DCI	6–8 weeks	TG, TC	26.5 ± 4.42, 28.2 ± 4.74	Decreased TG, TC
Nestler et al. [23]	1999	22/22	Venezuela/PCOS	1200 mg DCI	6–8 weeks	TG, TC, LDL-C, HDL-C	26 ± 5, 29 ± 6	Decreased TG

*MI* myo-inositol, *DCI* D-chiro-Inositol, *FA* folic acid, *HDL-C* high density lipoprotein-cholesterol, *LDL-C* low density lipoprotein-cholesterol, *MetS* metabolic syndrome, *PCOS* polycystic ovary syndrome, *TC* total cholesterol, *TG* triglycerides, *T2DM* type 2 diabetes mellitus

**Fig. 2:**
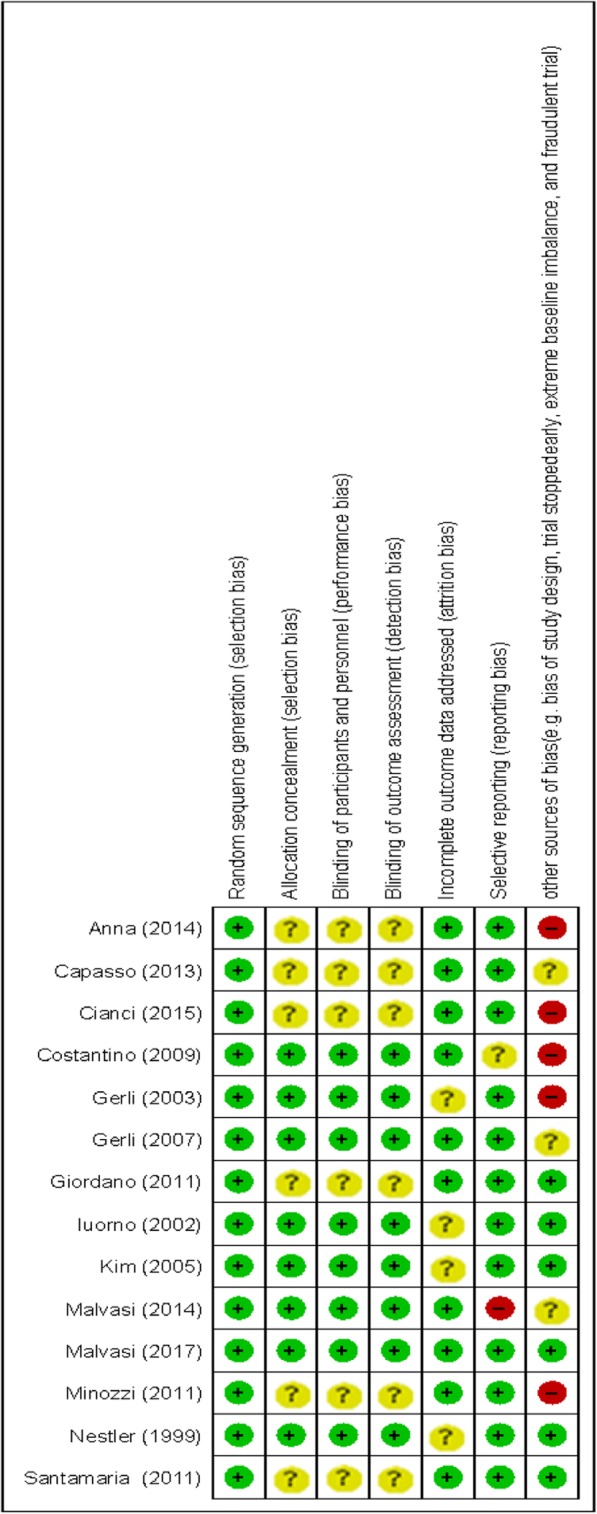
The methodological quality of included studies (risk of bias)

The quality assessment of the included studies, using the Cochrane Collaboration risk of bias tool is presented in Fig. 2.

**Fig. 3:**
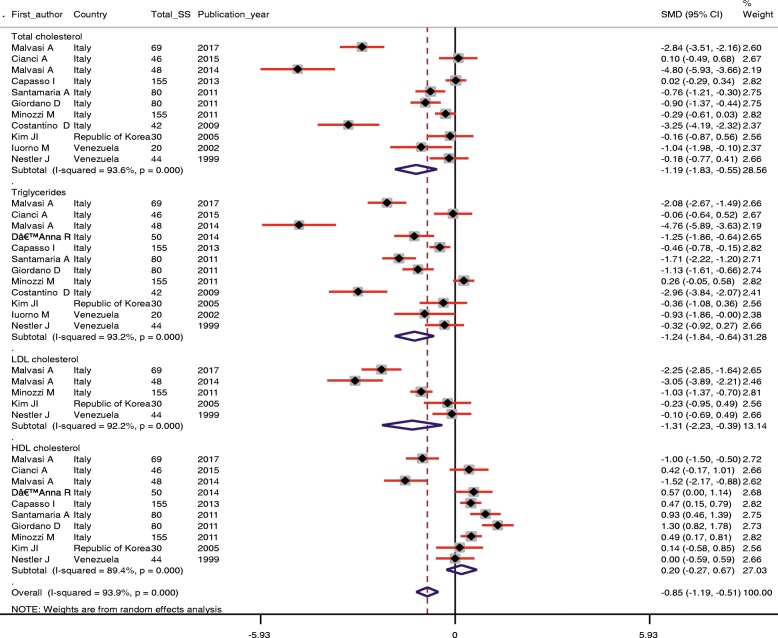
Meta-analysis lipid profiles standardized mean differences estimates for lipid profiles in inositol and placebo groups (CI = 95%)

Our meta-analysis findings showed that inositol supplementation among patients with metabolic diseases significantly decreased triglycerides (SMD − 1.24; 95% CI, − 1.84, − 0.64; *P* < 0.001), total- (SMD − 1.09; 95% CI, − 1.83, − 0.55; *P* < 0.001), and LDL-cholesterol levels (SMD − 1.31; 95% CI, − 2.23, − 0.39; *P* = 0.005) (Table 2 and Fig. 3). Inositol supplementation did not affect the HDL-cholesterol levels (SMD 0.20; 95% CI, − 0.27, 0.67; *P* = 0.40).

**Table 2:** Estimation of the standardized difference means of related indictors with CI 95% between the intervention and placebo groups

Variables		Number of study	Standardized mean difference	CI 95%	Heterogeneity		
					I-squared (%)	Q	*P*-value
Triglycerides	Intervention group (after vs. before)	12	− 1.84	− 2.62, − 1.06	95.6	248.31	< 0.001
	Placebo group (after vs. before)	12	− 0.17	− 0.39, 0.05	53.9	23.84	0.01
	Intervention group vs. placebo group	12	− 1.24	− 1.84, − 0.64	93.2	161.55	< 0.001
Total cholesterol	Intervention group (after vs. before)	11	− 1.40	− 2.11, − 0.69	94.7	189.67	< 0.001
	Placebo group (after vs. before)	11	0.22	−0.17, 0.61	84.7	65.30	< 0.001
	Intervention group vs. placebo group	11	−1.19	− 1.83, −0.55	93.6	156.53	< 0.001
LDL-cholesterol	Intervention group (after vs. before)	5	−1.28	−2.37, −0.18	94.4	71.88	< 0.001
	Placebo group (after vs. before)	5	0.23	0.02, 0.44	0.00	3.13	0.53
	Intervention group vs. placebo group	5	−1.31	−2.23, −0.39	92.2	51.00	< 0.001
HDL-cholesterol	Intervention group (after vs. before)	10	0.37	−0.13, 0.87	90.7	96.93	< 0.001
	Placebo group (after vs. before)	10	−0.02	− 0.20, 0.15	27.2	12.37	0.19
	Intervention group vs. placebo group	10	0.20	−0.27, 0.67	89.4	84.52	< 0.001

Because of the heterogeneity between included studies, we performed multiple subgroup analyses by suspected variables including type of disease, dosage of inositol, and duration of the study. The results demonstrated that the heterogeneity decreased in a number of subgroups, particularly in type of disease and the duration of study for triglycerides and HDL-cholesterol (Table 3).

**Table 3:** The association between inositol intake and lipid profiles based on subgroup analysis

Variables		Number of SMD included	Subgroups	Pooled OR (random effect)	95% CI	I^2^ (%)	Overall I^2^ (%)
Triglycerides	Type of disease	5	PCOS	−0.74	−1.66, 0.18	91.7	93.2
		7	Non-PCOS	−1.58	−2.32, −0.85	92.1	
	Dosage of inositol (mg/day)	6	≥2000	−0.83	−1.49, − 0.18	83.4	
		6	<2000	−1.69	−2.69, −0.68	96.2	
	Duration of study (week)	5	≥14	−1.64	−2.94, −0.34	93.4	
		7	<14	−0.99	−1.67, − 0.32	93.1	
	Type of intervention	4	DCI	−0.32	−0.66, 0.01	0.0	
		6	MI	−1.15	−1.91, −0.39	94.0	
		2	MI + DCI	−3.37	−6.00, −0.75	94.2	
Total cholesterol	Type of disease	5	PCOS	−0.86	−1.72, − 0.02	90.3	93.6
		6	Non-PCOS	−1.49	−2.51, −0.46	95.5	
	Dosage of inositol (mg/day)	5	≥2000	−0.82	−1.91, 0.28	92.2	
		6	<2000	−1.51	−2.38, −0.63	95.2	
	Duration of study (week)	5	≥14	−1.77	−3.31, −0.22	95.0	
		6	<14	−0.74	−1.33, − 0.15	90.5	
	Type of intervention	4	DCI	−0.22	−0.62, 0.18	26.2	
		5	MI	−0.91	−1.59, − 0.24	91.9	
		2	MI + DCI	−3.76	−5.68, −1.85	88.2	
LDL-cholesterol	Type of disease	3	PCOS	−0.60	−1.51, 0.31	86.1	92.2
		2	Non-PCOS	−1.84	−3.41, 0.26	93.1	
	Dosage of inositol (mg/day)	3	≥2000	−0.86	−2.27, 0.54	93.2	
		2	<2000	−2.00	−3.98, −0.03	94.8	
	Duration of study (week)	4	≥14	−1.39	−2.78, −0.01	94.0	
		1	<14	−1.03	− 1.37, −0.70	0.0	
	Type of intervention	2	DCI	−0.15	− 0.61, 0.30	0.0	
		1	MI	−1.03	−1.37, −0.70	–	
		2	MI + DCI	−2.59	−3.39, −1.81	56.7	
HDL-cholesterol	Type of disease	3	PCOS	0.38	0.12, 0.64	2.4	89.4
		7	Non-PCOS	0.14	−0.56, 0.84	92.7	
	Dosage of inositol (mg/day)	5	≥2000	0.01	−0.59, 0.62	81.1	
		5	<2000	0.37	−0.31, 1.04	92.2	
	Duration of study (week)	4	≥14	−0.61	−1.35, 0.14	83.5	
		6	<14	0.68	0.42, 0.95	55.1	
	Type of intervention	3	DCI	0.19	−0.17, 0.55	0.0	
		5	MI	0.72	0.42, 1.02	62.0	
		2	MI + DCI	−1.22	−1.73, −0.71	36.2	

*MI* myo-inositol, *DCI* D-chiro-Inositol, *PCOS* polycystic ovary syndrome

### Effects of inositol supplementation on triglycerides levels

In stratified analyses by inositol, the non-PCOS category had the strongest effect on reducing triglycerides levels (SMD: − 1.58; 95%CI: − 2.32, − 0.85; I^2^:92.1%) compared to the studies with PCOS patients (Table 3). In addition, in stratified analyses by the dosage of inositol, the <2000 mg/day category had the strongest effect on reducing triglycerides levels (SMD: − 1.69; 95% CI: − 2.94, − 0.34; I^2^:93.4%) compared to the ≥2000 mg/day category. In stratified analyses by the duration of study, the <14 weeks category had the strongest effect on reducing triglycerides levels (SMD: − 0.99; 95%CI: − 1.67, − 0.32; I^2^:93.1%) compared to the ≥14 weeks category. In stratified analyses by the type of interventions, the MI category (SMD: − 1.15; 95% CI, − 1.91, − 0.39; I^2^:94.0%) and MI plus DCI category (SMD − 3.37; 95% CI, − 6.00, − 0.75; I^2^:94.2%) had the strongest effect on reducing triglycerides levels compared with the DCI category.

### Effects of inositol supplementation on total cholesterol levels

In stratified analyses by inositol, the non-PCOS category had the strongest effect on decreasing total cholesterol concentrations (SMD: − 1.49; 95%CI: − 2.51, − 0.46; I^2^:95.5%) compared to the PCOS category (Table 3). In addition, in stratified analyses by the dosage of inositol, the <2000 mg/day category had the strongest effect on decreasing total cholesterol concentrations (SMD: − 1.51; 95% CI: − 2.38, − 0.63; I^2^:95.2%) compared to the ≥2000 mg/day category. In stratified analyses by the duration of study, the ≥14 weeks category had the strongest effect on decreasing total cholesterol concentrations (SMD: − 1.77; 95% CI: − 3.31, − 0.22; I^2^:95.0%) compared to the <14 weeks category. In stratified analyses by type of intervention, the MI category (SMD: − 0.91; 95% CI, − 1.59, − 0.24; I^2^:91.9%) and MI plus DCI category (SMD: − 3.76; 95% CI, − 5.68, − 1.85; I^2^:88.2%) had the strongest effect on reducing total cholesterol concentrations compared with the DCI category.

### Effects of inositol supplementation on LDL-cholesterol levels

In stratified analyses by the dosage of inositol, the <2000 mg/day category had the strongest effect on decreasing LDL-cholesterol levels (SMD: − 2.0; 95% CI: − 3.98, − 0.03; I^2^:94.8%) compared to the ≥2000 mg/day category (Table 3). In stratified analyses by the duration of study, the <14 weeks category had the strongest effect on decreasing LDL-cholesterol levels (SMD: − 1.03; 95%CI: − 1.37, − 0.70) compared to the ≥14 weeks category. LDL-cholesterol levels did not influence by type of diseases after inositol intake. In stratified analyses by type of intervention, the MI category (SMD: − 1.03; 95% CI, − 1.37, − 0.70) and MI plus DCI category (SMD: − 2.59; 95% CI, − 3.39, − 1.81; I^2^:56.7%) had the strongest effect on decreasing LDL-cholesterol levels compared with the DCI category.

### Effects of inositol supplementation on HDL-cholesterol levels

In stratified analyses by inositol, the PCOS category had the strongest effect on increasing HDL-cholesterol levels (SMD: 0.38; 95%CI: 0.12, 2.4; I^2^:2.4%) compared to the non-PCOS category (Table 3). In stratified analyses by the duration of study, the <14 weeks category had the strongest effect on increasing HDL-cholesterol levels (SMD: 0.68; 95%CI: 0.42, 0.95; I^2^:55.1%) compared to the ≥14 weeks category. HDL-cholesterol levels did not influence by dosage used after inositol intake. In stratified analyses by the type of intervention, the MI category had the strongest effect on increasing HDL-cholesterol levels (SMD: 0.72; 95% CI, 0.42, 1.02; I2:55.1%; I^2^:62.0%) compared with the DCI category. While, MI plus DCI category significantly decreased HDL-cholesterol (SMD: − 1.22; 95% CI, − 1.73, − 0.71; I^2^:36.2%).

### Sensitivity analysis and publication bias

**Fig. 4:**
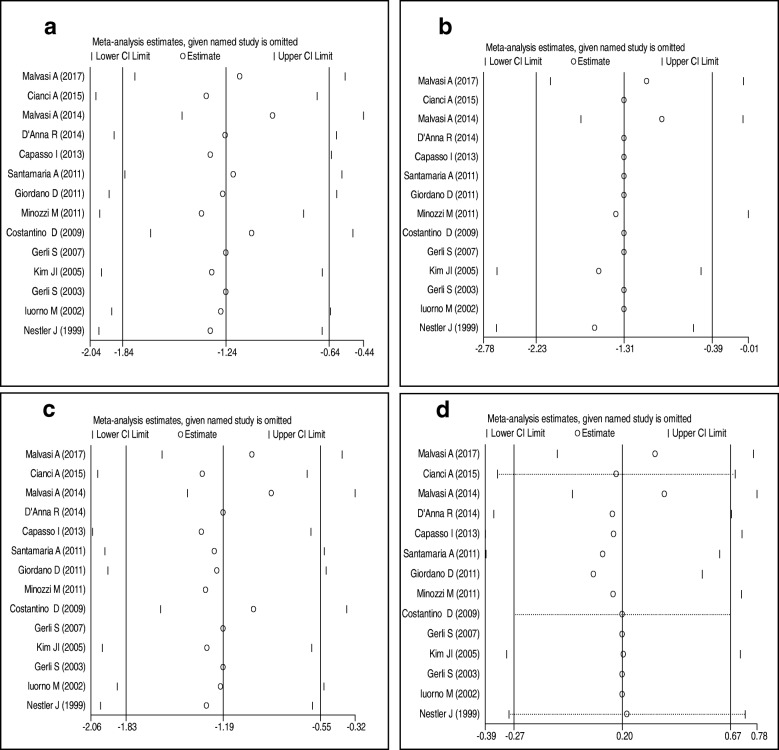
Sensitivity analysis inositol on lipid profiles; (**a**) triglycerides, (**b**) total cholesterol, (**c**) LDL-cholesterol, (**d**) and HDL-cholesterol for assess the effects of every study on pooled standardized mean differences estimates

Sensitivity analysis did not show any significant change regarding the effect of inositol on triglycerides, total-, LDL- and HDL-cholesterol after excluding each trial from meta-analysis one by one (Fig. 4).

There was no evidence of publication bias for meta-analyses assessing the effects of inositol on LDL- (B = − 2.63, *P* = 0.65), and HDL-cholesterol (B = − 3.90, *P* = 0.30) with Egger’s regression test.

There was evidence of publication bias on triglycerides (B = − 6.99, *P* = 0.01) and total cholesterol (B = − 6.87, *P* = 0.01), therefore we used non-parametric method (Duval and Tweedie) to estimate the results of censored studies. The meta-analysis based on these studies showed that summary effect size on total cholesterol was not significant between before (SMD − 1.19; 95% CI, − 1.83, − 0.55) included censored studies into meta-analysis and after (SMD − 1.33; 95% CI, − 2.00, − 0.66). In addition, summary effect size SMD of triglycerides increased form before (SMD − 1.24; 95% CI, − 1.84, − 0.64) to after (SMD − 1.54; 95% CI, − 2.30, − 0.79) included censored studies into meta-analysis.

## Discussion

To our knowledge, this is the first meta-analysis of RCTs that evaluated the effect of inositol supplementation on lipid profiles among populations with metabolic diseases. We found that inositol supplementation may result in an improvement in triglycerides, total- and LDL-cholesterol levels, but did not affect HDL-cholesterol levels among populations with metabolic diseases.

Therapeutic lifestyle changes aimed at pursuing an acceptable control of risk factors of metabolic disturbances, including dyslipidemia and hyperglycemia, are limited by poor adherence and persistence [29, 30]. A general consensus already exists on the first line approach in peoples with metabolic diseases which includes the lifestyle modifications, including diet, physical exercise and regular sleep pattern. However, we believe that implementation of behavioral changes cannot be easily reached by every patient and/or in all situation, resulting poor compliance. Since insulin resistance is the major driver of MetS, the use of insulin sensitizer is therefore well established, in order to decrease comorbidities that characterize MetS [31]. Some studies have demonstrated the beneficial effects of inositol supplementation on lipid profiles in patients with metabolic status; however, findings are controversial. For example, Kim et al. [21] demonstrated that inositol supplementation for 13 weeks to patients with T2DM significantly decreased total-, LDL-, LDL/HDL-cholesterol ratio, and significantly increased HDL-cholesterol, but did not affect triglycerides levels. In addition, inositol supplementation at a dosage of 1200 mg/day for 6 to 8 weeks among obese women with PCOS improved the action of insulin, ovulatory function, androgen levels, blood pressures, and triglycerides levels [23]. A significant reduction in triglycerides (− 43.2%) and a significant increase in HDL-cholesterol levels (48.6%) were also evidenced following the supplementation with inositol for 6 months in postmenopausal women with MetS, but did not affect other lipid profiles [25]. In a meta-analysis study by Pundir et al. [15], inositols significantly improved menstrual cycles, ovulation and metabolic changes in PCOS. Irrespective of the speculative assumptions on the possible beneficial effects of triglycerides and HDL-cholesterol increase, there is substantial agreement that high triglycerides, total- and LDL-cholesterol, and low HDL-cholesterol concentrations have a detrimental influence on CVD prognosis [32, 33]. In addition, previous studies have reported that nutraceuticals play a peculiar role in ameliorating human dyslipidaemia [34, 35], which in turn effectively able to reduce the burden of the atherosclerosis process and the progress of CVD [36]. Nutraceuticals may improve lipid profiles through the upregulation of hepatic LDL receptors, decrease in the intestinal absorption of cholesterol [37], blocking carbohydrate digestion and glucose absorption in the gut, decreasing glucose release from liver, and activating insulin receptors, and glucose uptake in insulin-sensitive tissues [38].

In this meta-analysis study, inositol supplementation had benefit on HDL-cholesterol levels in patients with PCOS; however, it should no effect on HDL-cholesterol levels in non-PCOS patients. Duration of study in the included trials was varied from 6 weeks to 12 months; in our subgroup analysis, duration of study <14 weeks was compared with ≥14 weeks of inositol supplementation and showed a significantly beneficial effect on increasing circulating HDL-cholesterol levels with a longer duration of supplementation.

It has been suggested that the binding of insulin to specific receptors stimulates transport of inositol phosphoglycan intracellularly and explains its role as a mediator in the insulin signaling cascade [39]. Furthermore, decreasing insulin resistance after the intake of inositol may be due to an improvement in peripheral insulin sensitivity [40]. Decreasing insulin resistance may improve lipid profiles. In addition, inositol intake may improve lipid metabolism through lowering visceral fat weight, hepatic lipid accumulation and insulin secretion as well as by increasing adiponectin concentrations [41]. Adipocytokine concentrations are associated with insulin sensitivity and resistance [42]. Adiponectin is the most important factor for increasing insulin sensitivity, while factors including leptin, resistin and C-reactive protein are known to be correlated with the increase of insulin resistance [43]. In a study, MI supplementation (4 g/day) for 8 weeks among patients with gestational diabetes significantly increased adiponectin levels [13]. In another study, co-supplementation with MI, soy isoflavones and cocoa polyphenols for 6 months among postmenopausal women with MetS significantly resistin levels [24]. Furthermore, a significant weight loss and leptin reduction following the administration of MI [20] my result in an improvement in the lipid profiles.

The current study had a few limitations. Various doses and different types of inositol were administered for intervention in the included studies. We were unable to assess the dose response association between supplementation and lipid profiles. There was high heterogeneity among included studies in our meta-analysis; however, heterogeneity decreased after sub-group analysis based on type of intervention. Our results should be interpreting with more caution.

## Conclusions

Inositol supplementation may result in reduction in triglycerides, total- and LDL-cholesterol levels, but did not affect HDL-cholesterol levels among patients with metabolic diseases. Additional prospective studies regarding the effect of inositol supplementation on lipid profiles in patients with metabolic diseases are necessary.

## Abbreviations

DCI: D-chiro-Inositol
FA: Folic acid
HDL-C: High density lipoprotein-cholesterol
LDL-C: Low density lipoprotein-cholesterol
MetS: Metabolic syndrome
MI: Myo-inositol
PCOS: Polycystic ovary syndrome
T2DM: Type 2 diabetes mellitus
TC: Total cholesterol
TG: Triglycerides
